# The Etiology of Intraocular Lens Dislocation and Changes in Intraocular Pressure After Intrascleral Intraocular Lens Fixation Surgery

**DOI:** 10.7759/cureus.75295

**Published:** 2024-12-07

**Authors:** Yuya Saito, Norihiro Shimizu, Yoichi Mashimo, Tomoaki Tatsumi, Hirotaka Yokouchi, Takayuki Baba

**Affiliations:** 1 Ophthalmology, Asahi General Hospital, Chiba, JPN; 2 Ophthalmology and Visual Science, Chiba University Graduate School of Medicine, Chiba, JPN; 3 Ophthalmology, Maebara Shimizu Eye Clinic, Funabashi, JPN; 4 Public Health, Chiba University Graduate School of Medicine, Chiba, JPN; 5 Ophthalmology, Teikyo University Chiba Medical Center, Ichihara, JPN

**Keywords:** ectopia lentis, etiology, iol intrascleral fixation, iop change, lens dislocation

## Abstract

Objectives

This study aimed to identify the etiology and the direction of dislocation of the natural crystalline lens or intraocular lens (IOL) in IOL intrascleral fixation surgery and to determine the change in intraocular pressure (IOP) after surgery.

Methods

We retrospectively investigated the diagnosis, direction of lens and IOL dislocation, and IOP before and after surgery (preoperatively and one day, one week, and one month postoperatively) in 236 eyes from 228 patients who underwent IOL intrascleral fixation at Chiba University Hospital between February 2015 and September 2020.

Results

IOL intrascleral fixation was performed in 48 (20.3%) patients with long eye axis, 44 (18.6%) with pseudoexfoliation (PEX), 42 (17.8%) with intraoperative problems such as ciliary zonule rupture or posterior capsule rupture, 40 (16.9%) with a history of trauma, 34 (14.4%) with a history of vitrectomy, 21 (8.9%) with atopic dermatitis, six (2.5%) with genetic diseases such as Marfan syndrome, four (1.7%) with retinitis pigmentosa, and 58 (24.6%) with unknown causes. Downward IOL dislocation was the most common (52 cases), while IOL falling into the vitreous cavity was seen in 46 cases, aphakic eye in 31 cases, and anterior lens dislocation in 16 cases. In general, IOP was significantly lower at one month postoperatively than preoperatively. Specifically, in the trauma, PEX, and unknown causes groups, as well as the groups with anterior lens deviation, IOL fell into the vitreous cavity, and with IOL downward deviation, IOP was significantly lower.

Conclusion

IOP was significantly lower one month postoperatively than preoperatively after IOL intrascleral fixation. Patients who underwent IOL intrascleral fixation surgery were most commonly found to have long eye axis, PEX, and intraoperative problems. Downward deviation of the IOL or IOL falling into the vitreous cavity was most common. IOP was significantly lower one month postoperatively than preoperatively after IOL intrascleral fixation.

## Introduction

Various surgical techniques have been devised to fix an intraocular lens (IOL) in the eye when IOL insertion is not possible due to crystalline lens dislocation/subluxation (ectopia lentis), IOL dislocation/subluxation, lens capsule breakage, or rupture of the ciliary zonule [1]. However, there have been several problems with previous techniques. For example, the insertion of an IOL into the anterior chamber is associated with a high risk of corneal endothelial damage and bullous keratopathy [2]. Flanged IOL fixation with the double-needle technique solves many of these problems and has been widely used in recent years as it is safe and simple [1].

The causes for IOL intrascleral fixation include acquired breakage of the ciliary zonule due to trauma, history of vitrectomy, problems during cataract surgery (such as posterior capsule rupture), and internal factors (pseudoexfoliation {PEX} syndrome and genetic diseases), but few reports have reviewed these factors in detail, especially from the points of the direction of dislocation, etiology, and changes in intraocular pressure in Asia [3-6]. It has been reported that intraocular pressure (IOP) in patients with PEX can be lowered by treatment of IOL dislocation [4-7]. A high prevalence of zonular instability has been reported in retinitis pigmentosa (RP) patients [8].

In patients with atopic dermatitis, it has been noted that the ciliary zonules are fragile and torn due to the daily application of chronic blunt stimuli such as facial scraping and punching. In addition, recent reports of lens epithelial cell degeneration and increased fragility of the lens capsule have occurred in patients with atopic dermatitis [9,10].

The purpose of this study is to retrospectively examine patients who have undergone IOL intrascleral fixation surgery at our institution to identify single and multiple predisposing conditions that may contribute to abnormal lens and IOL position. In addition, the directionality of dislocation will be assessed and pre and postoperative IOL pressure records, if available, will be compared. Thus, this study aimed to identify the etiology and direction of dislocation of the natural crystalline lens or IOL in IOL intrascleral fixation surgery and the change in IOP after surgery.

## Materials and methods

**Figure 1:**
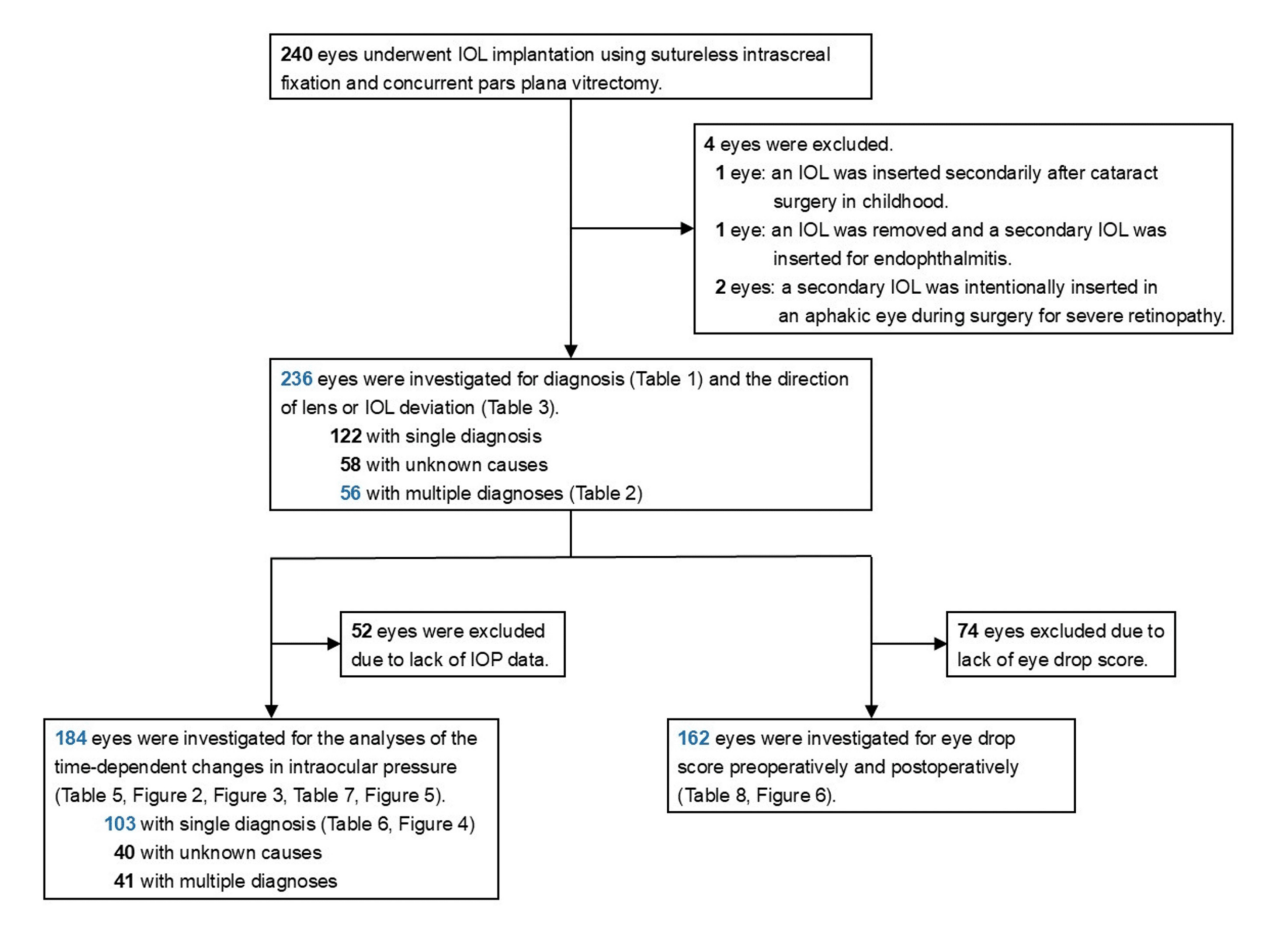
Study participant eligibility criteria.

To clarify the above issues, we retrospectively conducted the investigation shown in Figure 1. Medical records of 240 eyes (232 patients) that underwent IOL implantation using sutureless intrascleral fixation and concurrent pars plana vitrectomy between February 2015 and September 2020 at Chiba University Hospital were retrospectively reviewed. Intrascleral fixation was performed using the flanged intrascleral intraocular lens fixation technique using the double-needle method published by Yamane et al. [1]. Two incisions were made with a 30-gauge needle, the IOL haptics was fitted with the needle, and the tip was cauterized to create a flange for the haptics. The flange of the haptics was pushed back and fixed in the scleral tunnel. Four cases were excluded from this study because of the following reasons: one case in which an IOL was inserted secondarily after cataract surgery in childhood, one case in which an IOL was removed and a secondary IOL was inserted for endophthalmitis, and two cases in which a secondary IOL was intentionally inserted in an aphakic eye during surgery for severe retinopathy.

All data (age, sex, diagnoses, eye drop scores, IOP, and direction of lens or IOL deviation) were collected from medical records. The direction of deviation was determined by the position of the lens or IOL when the patient was examined in the sitting position in front of the slit lamp. The direction of deviation was classified as "aphakia," "vitreous cavity," "upward," "downward," "temporal," "nasal," or "anterior." There were no cases with retinotopic deviation from the pupillary plane that did not fall into the vitreous cavity, nor were there any cases with nasal deviation recorded. In this context, "aphakia" refers to the absence of the lens and IOL in the eye at the time of IOL intrascleral fixation surgery. These are cases in which IOL implantation was not performed due to problems during cataract surgery or cases in which IOL insertion was intentionally not performed during previous vitrectomy for endophthalmitis or proliferative retinopathy. Upward means that the lens or IOL was deviated toward the head, downward means toward the foot, and anterior means that the lens or IOL was deviated toward the cornea from the pupillary plane.

Candidate diagnoses were listed as follows based on previous reports: (1) PEX, (2) trouble in surgery (intraoperative problems during cataract surgery, such as rupture of the ciliary zonule or posterior capsule cases), (3) history of trauma, (4) genetic diseases (such as Marfan syndrome), (5) history of vitrectomy, (6) retinitis pigmentosa (RP), (7) history of atopic dermatitis, and (8) long eye axis (>26 mm) [3,7].

Based on the medical records, the presence of these diagnoses was checked for each case. Cases with more than one diagnosis were counted separately for each diagnostic group. Eye drop scores were examined with regard to glaucoma medication use before and after surgery. The eye drop score was 1 point per glaucoma eye drop (2 points for combination drugs). Acetazolamide oral medication was scored as 1 point.

IOP was measured with a noncontact tonometer (NCT) using a TONOREF II auto-refractometer (Aichi, Japan: Nidek) or a Goldmann applanation tonometer (GAT) (Jena, Germany: Carl Zeiss AG) preoperatively and at one day, one week, and one month after surgery. In cases where IOP is measured by GAT, GAT is preferred to NCT. In addition, in this institution, the day after surgery or if the IOP is abnormal, it is measured by GAT. Fifty-two cases with a lack of some IOP data were excluded from further IOP analysis. The majority of excluded cases were referred to an introduction origin medical institution within the first postoperative month and we were unable to measure IOP.

Statistical analyses were performed with the Mann-Whitney U test and mixed-effect model using R version 4.1.2 (https://www.r-project.org/) (Vienna, Austria: The R Foundation). A mixed-effect model analysis (subject as a random effect) was conducted using the lme4 package (https://cran.r-project.org/web/packages/lme4/index.html) and lmerTest (https://cran.r-project.org/web/ packages/lmerTest/index.html) to compare the preoperative and postoperative IOP. Statistical significance was set at p<0.05.

The study protocol was approved by the Institutional Review Board of Chiba University Graduate School of Medicine (#3917) and the procedures complied with the tenets of the Declaration of Helsinki. Patients were informed about the purpose of the study via the Internet and were allowed to opt out of the study. Data collection was conducted based on the Ethical Guidelines for Medical and Health Research Involving Human Subjects in Japan (https://www.mhlw.go.jp/file/06-Seisakujouhou-10600000-Daijinkanboukouseikagakuka/0000080278.pdf).

This article was previously published as a preprint on the Research Square server on November 16, 2022.

## Results

The diagnoses of a total of 236 cases (160 male and 76 female patients) with a median age of 68 years are summarized in Table 1. The most common diagnosis was long eye axis (n=48, 20.3%), followed by PEX (n=44, 18.6%), trouble in surgery (n=42, 17.8%), history of trauma (n=40, 16.9%), history of vitrectomy (n=34, 14.4%), history of atopic dermatitis (n=21, 8.9%), genetic disease (n=6, 2.5%), and RP (n=4, 1.7%). In addition, 58 cases (24.6%) had unknown causes, and 56 cases (23.7%) had multiple diagnoses. In the 56 cases with multiple diagnoses listed in Table 2, the most common combination of diagnoses was PEX and trouble in surgery (n=18), followed by long eye axis and history of vitrectomy (n=15).

**Table 1:** Diagnoses of cases undergone intrascleral intraocular lens fixation surgery. ^*^Cases with multiple diagnoses were counted separately for each diagnostic group. The data were represented as n(%), for the observed number of cases (case or sex), median, and IQR for age. IQR: interquartile range; PEX: pseudoexfoliation; RP: retinitis pigmentosa

Diagnosis		Cases, n (%)	Age, median (IQR)	Sex	
				Male, n (%)	Female, n (%)
Diagnosis group*	PEX	44 (18.6)	83 (77-85)	22 (13.8)	22 (28.9)
	Trouble in surgery	42 (17.8)	78.5 (73.25-84.75)	21 (13.1)	21 (27.6)
	Trauma	40 (16.9)	65.5 (55-72.25)	33 (20.6)	7 (9.2)
	Genetic disease	6 (2.5)	47 (43.5-59.5)	3 (1.9)	3 (3.9)
	Vitrectomy	34 (14.4)	64.5 (53-71.75)	28 (17.5)	6 (7.9)
	RP	4 (1.7)	65.5 (58-68.5)	0 (0)	4 (5.3)
	Atopic dermatitis	21 (8.9)	48 (42-49)	16 (10)	5 (6.6)
	Long eye axis (>26 mm)	48 (20.3)	58 (51.75-67.75)	38 (23.8)	10 (13.2)
	Unknown causes	58 (24.6)	66.5 (60-72.75)	40 (25)	18 (23.7)
Multiple diagnoses		56 (23.7)	65.5 (54-77.5)	38 (23.8)	18 (23.7)
Total		236	68 (56-78)	160	76

**Table 2:** Number of the cases with multiple diagnoses. ^*^The cases with long eye axis and history of vitrectomy (n=15). ^**^The cases with PEX and trouble in surgery (n=18). The data were represented as "n" for the observed number of cases. PEX: pseudoexfoliation; RP: retinitis pigmentosa

Combination of diagnoses			Cases, n
1	2	3	Total (n=56)
Long eye axis (>26 mm)	Trauma	-	6
Long eye axis (>26 mm)	Trouble in surgery	-	3
Long eye axis (>26 mm)	Genetic disease	-	1
Long eye axis (>26 mm)	Atopic dermatitis	-	5
Long eye axis (>26 mm)	RP	-	1
Long eye axis (>26 mm)	Vitrectomy	-	11^*^
Long eye axis (>26 mm)	Vitrectomy	Trauma	1^*^
Long eye axis (>26 mm)	Vitrectomy	Genetic disease	1^*^
Long eye axis (>26 mm)	Vitrectomy	Atopic dermatitis	1^*^
Long eye axis (>26 mm)	PEX	Vitrectomy	1^*^
Long eye axis (>26 mm)	PEX	Trouble in surgery	1^**^
PEX	Trouble in surgery	-	17^**^
PEX	Trauma	-	1
PEX	Vitrectomy	-	1
Trauma	Atopic dermatitis	-	1
Trauma	Vitrectomy	-	1
Trouble in surgery	Atopic dermatitis	-	1
Vitrectomy	Atopic dermatitis	-	1
Vitrectomy	RP	-	1

The directions of lens and IOL deviation of the 236 cases were as follows: 31 (13.1%) were aphakic eyes; 12 (5.1%) with the natural crystalline lens displaced into the vitreous cavity, four (1.7%) with the natural crystalline lens deviation upward, one (0.4%) nasally, eight (3.4%) downward, two (0.8%) temporal, and 16 (6.8%) anteriorly; 46 (19.5%) with the IOL displaced into the vitreous cavity, and six (2.5%) with the IOL deviation upward, one (0.4%) nasally, 52 (22.0%) downward, four (1.7%) temporal, six (2.5%) cases anteriorly, and one (0.4%) posteriorly; and 46 cases (19.5%) showed no evidence of deviation (Table 3). Fifteen of these patients were converted to IOL intrascleral fixation during cataract surgery at our institution. In addition, 25 and five of the 46 cases had phacodonesis of the natural crystalline lens and IOL, respectively.

**Table 3:** Direction of lens and intraocular lens deviation of cases undergone intrascleral intraocular lens fixation surgery. The data were represented as n (%), for the observed number of cases (case or sex), median, and IQR for age. IQR: interquartile range; IOL: intraocular lens

Direction of lens or IOL deviation		Cases, n (%)	Age, median (IQR)	Sex	
				Male, n (%)	Female, n (%)
Aphakia		31 (13.1)	77 (71.5-81.5)	20 (12.5)	11 (14.5)
Crystalline lens	Vitreous cavity	12 (5.1)	69.5 (62.75-73.75)	9 (5.6)	3 (3.9)
	Upward	4 (1.7)	65 (53.5-73.75)	2 (1.3)	2 (2.6)
	Nasal	1 (0.4)	62	1 (0.6)	0 (0)
	Downward	8 (3.4)	67 (58.5-69.25)	3 (1.9)	5 (6.6)
	Temporal	2 (0.8)	60.5 (51.75-69.25)	2 (1.3)	0 (0)
	Anterior	16 (6.8)	59 (53.75-71.75)	9 (5.6)	7 (9.2)
IOL	Vitreous cavity	46 (19.5)	62 (53-70.75)	38 (23.8)	8 (10.5)
	Upward	6 (2.5)	54 (46-62)	5 (3.1)	1 (1.3)
	Nasal	1 (0.4)	53	1 (0.6)	0 (0)
	Downward	52 (22)	70.5 (59.5-81.5)	35 (21.9)	17 (22.4)
	Temporal	4 (1.7)	53.5 (47-63.75)	4 (2.5)	0 (0)
	Anterior	6 (2.5)	60.5 (51.75-68.5)	4 (2.5)	2 (2.6)
	Posterior	1 (0.4)	44	0 (0)	1 (1.3)
No dislocation		46 (19.5)	71.5 (62.25-84)	27 (16.9)	19 (25)

In the PEX and trouble in surgery groups, the cases with aphakic eyes (PEX: 16 of 44, trouble in surgery: 23 of 42) and no deviation (PEX: 12 of 44, trouble in surgery: 15 of 42) were observed mainly, whereas the cases with the IOL fall into the vitreous cavity (vitrectomy: 20 of 34, long eye axis: 19 of 48) and the IOL deviation downward (vitrectomy: 10 of 34, long eye axis: 13 of 48) were observed in the history of vitrectomy and long eye axis groups (Table 4). The cases with the deviation in various directions were observed in the history of trauma group, and five of the 16 cases with the natural crystalline lens deviation anteriorly had a history of trauma.

**Table 4:** Number of cases by diagnosis group and direction of lens/intraocular lens deviation. The data were represented as n for the observed number of cases. Number after excluding the cases with multiple diagnoses is indicated in parentheses. PEX: pseudoexfoliation; RP: retinitis pigmentosa; IOL: intraocular lens

Direction of lens or IOL deviation		Diagnosis group										
		PEX	Trouble in surgery	Trauma	Genetic disease	Vitrectomy	RP	Atopic dermatitis	Long eye axis (>26 mm)	Unknown causes	PEX + trouble in surgery	Vitrectomy + long eye axis (>26 mm)
Aphakia		16 (3)	23 (8)	3 (3)	0 (0)	1 (0)	0 (0)	0 (0)	4 (0)	1	13	1
Crystalline lens	Vitreous cavity	0 (0)	0 (0)	5 (5)	0 (0)	0 (0)	0 (0)	0 (0)	2 (2)	5	0	0
	Upward	0 (0)	0 (0)	2 (2)	0 (0)	0 (0)	0 (0)	0 (0)	0 (0)	2	0	0
	Nasal	0 (0)	0 (0)	1 (1)	0 (0)	0 (0)	0 (0)	0 (0)	0 (0)	0	0	0
	Downward	2 (2)	0 (0)	2 (2)	3 (3)	0 (0)	0 (0)	0 (0)	0 (0)	1	0	0
	Temporal	0 (0)	0 (0)	1 (1)	1 (1)	0 (0)	0 (0)	0 (0)	0 (0)	0	0	0
	Anterior	3 (2)	1 (0)	5 (5)	0 (0)	0 (0)	1 (1)	0 (0)	2 (2)	5	1	0
IOL	Vitreous cavity	2 (0)	1 (0)	5 (1)	1 (0)	20 (10)	1 (0)	10 (6)	19 (4)	7	0	8
	Upward	0 (0)	1 (1)	0 (0)	0 (0)	1 (0)	0 (0)	1 (0)	2 (1)	3	0	1
	Nasal	0 (0)	0 (0)	0 (0)	0 (0)	1 (0)	0 (0)	0 (0)	1 (0)	0	0	1
	Downward	9 (8)	1 (1)	7 (4)	1 (0)	10 (4)	1 (1)	3 (2)	13 (6)	17	0	4
	Temporal	0 (0)	0 (0)	0 (0)	0 (0)	0 (0)	0 (0)	1 (1)	0 (0)	3	0	0
	Anterior	0 (0)	0 (0)	0 (0)	0 (0)	0 (0)	0 (0)	1 (0)	1 (0)	5	0	0
	Posterior	0 (0)	0 (0)	0 (0)	0 (0)	0 (0)	0 (0)	0 (0)	1 (1)	0	0	0
No dislocation		12 (8)	15 (10)	9 (6)	0 (0)	1 (1)	1 (0)	5 (3)	3 (0)	9	4	0
Total		44 (23)	42 (20)	40 (30)	6 (4)	34 (15)	4 (2)	21 (12)	48 (16)	58	18	15

**Figure 2:**
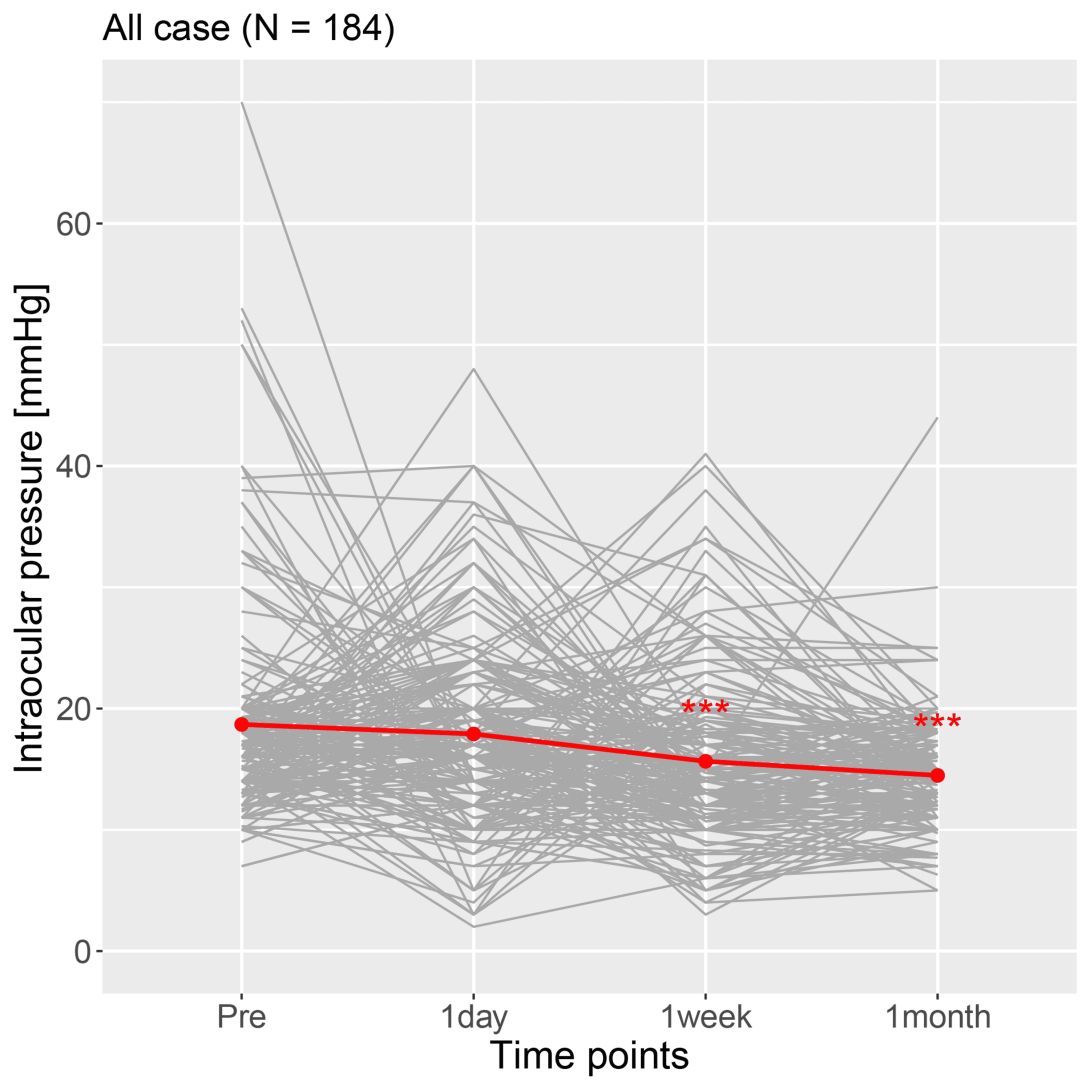
The time course changes in IOP in 184 individual cases. IOP: intraocular pressure

The time course changes in IOP in 184 individual cases are shown in Figure 2. The mean IOP at all time points (preoperative and one day, one week and one month postoperatively) in the 184 cases were 18.7±8.7 mmHg and 17.9±7.7 mmHg, 15.6±7.0 mmHg and 14.5±4.5 mmHg, respectively, (Table 5 and Figure 2, red line). One week and one month postoperatively IOPs showed a significant decrease from preoperative IOP (p<0.001, mixed-effect model).

**Table 5:** Intraocular pressure by diagnosis group of cases who underwent intrascleral intraocular lens fixation surgery. ^*^Cases with multiple diagnoses were counted separately for each diagnostic group. ^**^The p-values were calculated by repeated measures analysis of variance (ANOVA) using a mixed-effect model (random intercept) for the IOPs. ^***^A p-value <0.05 was considered statistically significant. The data were represented as n (%), for the observed number of cases (case or sex), median, and IQR for age and mean±SD for IOP (mmHg). IQR: interquartile range; IOP: intraocular pressure; SD: standard deviation; NumDF: numerator degrees of freedom; DenDF: denominator degrees of freedom; PEX: pseudoexfoliation; RP: retinitis pigmentosa; Pre: before surgery; After 1 day: 1 day after surgery; After 1 week: 1 week after surgery; After 1 month: 1 month after surgery

Diagnosis		Cases, n (%)	Age, median (IQR)	Sex		IOP (mmHg), mean±SD				NumDF**	DenDF**	F-value**	p-Value**
				Male, n	Female, n	Pre	After 1 day	After 1 week	After 1 month				
Diagnosis group*	PEX	34 (18.5)	83.5 (79.25-85)	18	16	18.5±8.0	17.3±6.7	16.5±7.7	15.9±6.6	3	99	1.19	0.317
	Trouble in surgery	30 (16.3)	77.5 (70.75-85)	17	13	15.9±5.6	19.2±7.2	15.6±7.9	15.5±6.8	3	87	2.41	0.073
	Trauma	33 (17.9)	66 (56-73)	26	7	20.4±10.0	18.2±9.1	15.5±6.1	14.4±3.7	3	96	4.99	0.003***
	Genetic disease	6 (3.3)	47 (43.5-59.5)	3	3	15.3±2.2	14.8±3.1	13.1±4.3	13.5±5.0	3	15	1.47	0.263
	Vitrectomy	25 (13.6)	63 (53-72)	20	5	18.2±4.4	15.4±5.3	17.4±6.9	14.5±4.1	3	72	3.15	0.030***
	RP	4 (2.2)	65.5 (58-68.5)	0	4	23.8±17.8	20.0±9.4	10.4±4.4	13.3±2.9	3	9	1.38	0.311
	Atopic dermatitis	19 (10.3)	48 (42-51.5)	15	4	20.7±8.5	18.1±6.3	17.3±6.3	15.2±4.4	3	54	2.73	0.053
	Long eye axis (>26 mm)	39 (21.2)	60 (52.5-72)	30	9	20.4±8.7	16.2±7.9	16.2±7.2	14.8±3.9	3	114	4.82	0.003***
	Unknown causes	40 (21.7)	68 (58.75-72.25)	25	15	18.6±10.0	19.4±8.2	15.5±7.1	13.7±3.0	3	117	5.25	0.002***
Multiple diagnoses		41 (22.3)	65 (54-76)	29	12	19.2±7.1	17.2±6.7	17.1±7.0	15.7±5.9	3	120	1.99	0.119
Total		184	68 (55-78)	122	62	18.7±8.7	17.9±7.7	15.6±7.0	14.5±4.5	3	549	16.2	<0.001***

**Figure 3:**
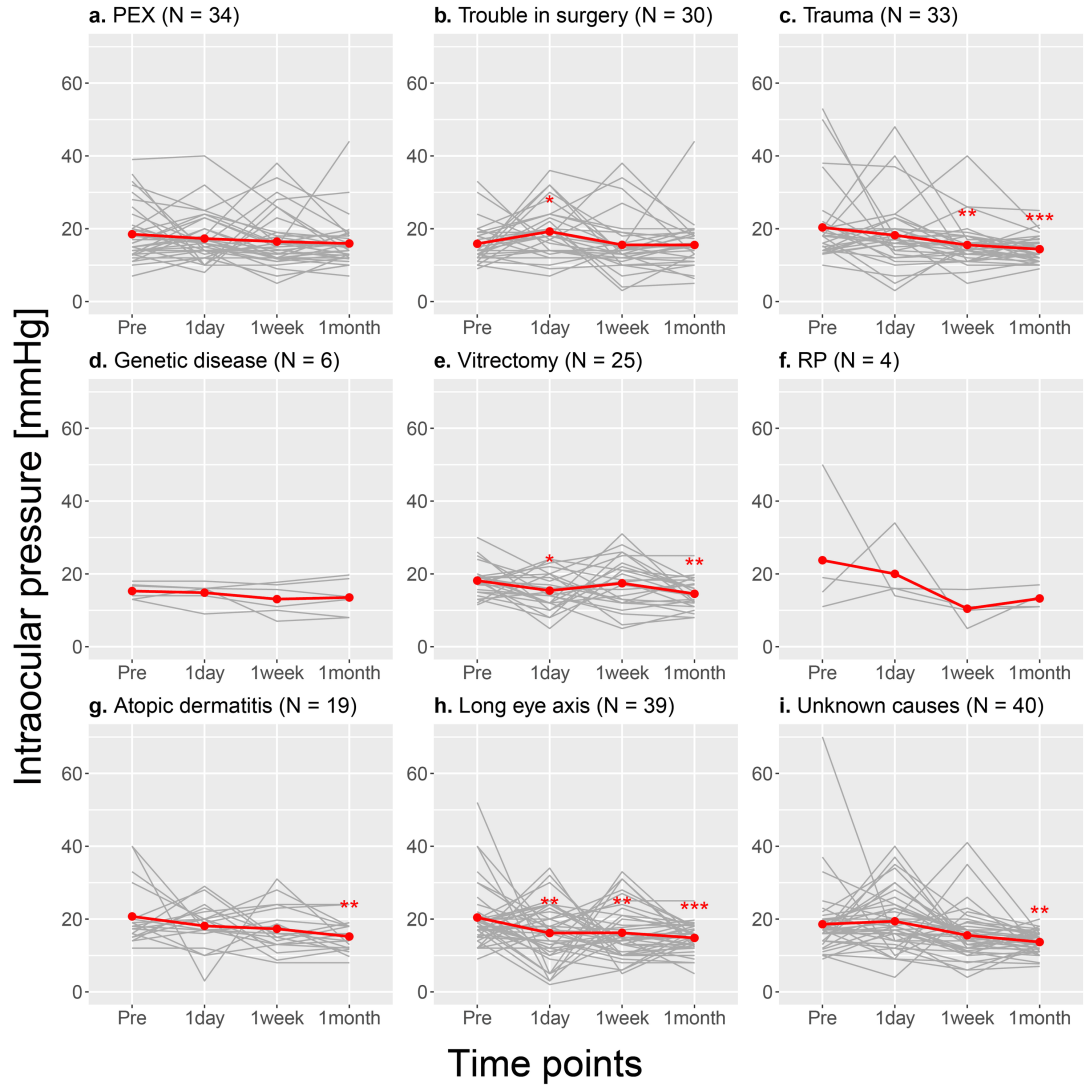
Changes in intraocular pressure preoperatively, one week and one month postoperatively for each diagnostic group. PEX: pseudoexfoliation; RP: retinitis pigmentosa

The mixed-effects model analysis for each diagnostic group showed significant decreases in IOPs at one week and one month postoperatively compared to preoperative values in the history of trauma and long eye axis groups. Additionally, the decrease in IOP at one month postoperatively was also significant in the history of vitrectomy, history of atopic dermatitis, and unknown causes groups (Table 5 and Figure 3).

**Figure 4:**
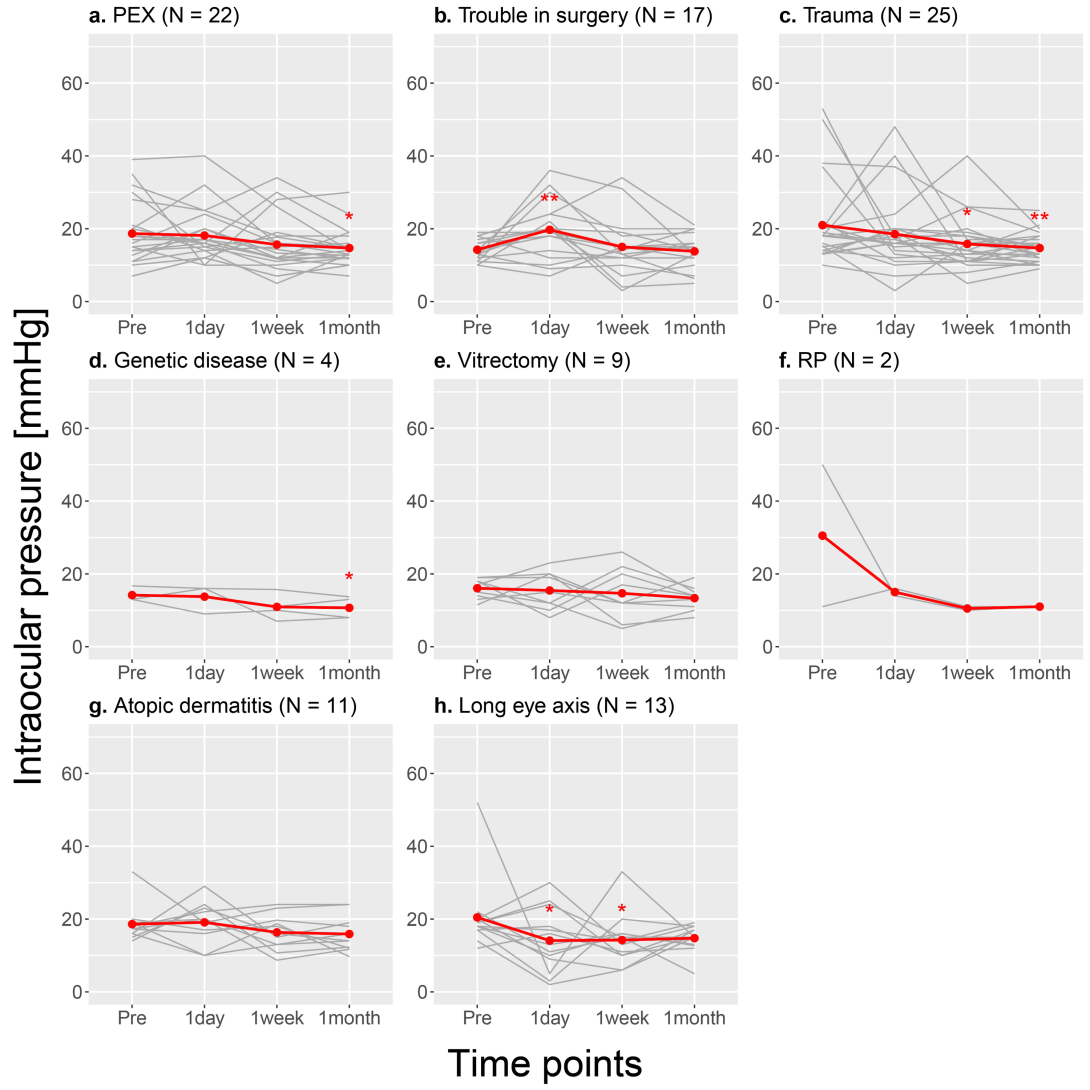
Changes in intraocular pressure preoperatively, one week and one month postoperatively for each diagnostic group (excluding the cases with multiple diagnoses). PEX: pseudoexfoliation; RP: retinitis pigmentosa

After excluding the cases with multiple diagnoses, the decrease of one month postoperative IOP in the history of trauma group was still significant, while that in the long eye axis group was not significant (Table 6 and Figure 4).

**Table 6:** Intraocular pressure by diagnosis group of cases who underwent intrascleral intraocular lens fixation surgery excluding the cases with multiple diagnoses. *The p-value in repeated measures analysis of variance (ANOVA) using mixed-effect model. **P<0.05 was considered significant. The data were represented as n(%), for the observed number of cases (case or sex), median, IQR for age; and mean±SD for IOP (mmHg). IQR: interquartile range; IOP: intraocular pressure; SD: standard deviation; NumDF: numerator degrees of freedom; DenDF: denominator degrees of freedom; PEX: pseudoexfoliation; RP: retinitis pigmentosa; Pre: before surgery; After 1 day: 1 day after surgery; After 1 week: 1 week after surgery; After 1 month: 1 month after surgery

Diagnosis group	Case, n	Age, median (IQR)	Sex		IOP (mmHg), mean±SD				p-Value*
			Male, n	Female, n	Pre	After 1 day	After 1 week	After 1 month	
PEX	22	83.5 (80-86.75)	12	10	18.7±8.9	18.1±7.3	15.6±7.7	14.7±5.0	0.034
Trouble in surgery	17	75 (73-81)	10	7	14.2±3.1	19.7±8.0	15.0±8.1	13.8±4.8	0.011**
Trauma	25	67 (56-72)	18	7	21.0±11.2	18.5±9.9	15.8±7.0	14.7±4.0	0.021**
Genetic disease	4	44 (43-49.5)	3	1	14.2±1.7	13.8±3.3	10.9±3.6	10.7±3.1	0.079
Vitrectomy	9	67 (64-76)	8	1	16.1±2.8	15.4±5.2	14.7±7.1	13.3±3.3	0.657
RP	2	55.5 (49.25-61.75)	0	2	30.5±27.6	15.0±1.4	10.5±0.7	11.0±0.0	0.525
Atopic dermatitis	11	47 (42-48)	7	4	18.6±5.1	19.1±5.7	16.3±4.9	15.9±4.9	0.251
Long eye axis (>26 mm)	13	59 (52-73)	10	3	20.5±9.8	14.1±8.7	14.2±6.9	14.7±3.6	0.104

**Figure 5:**
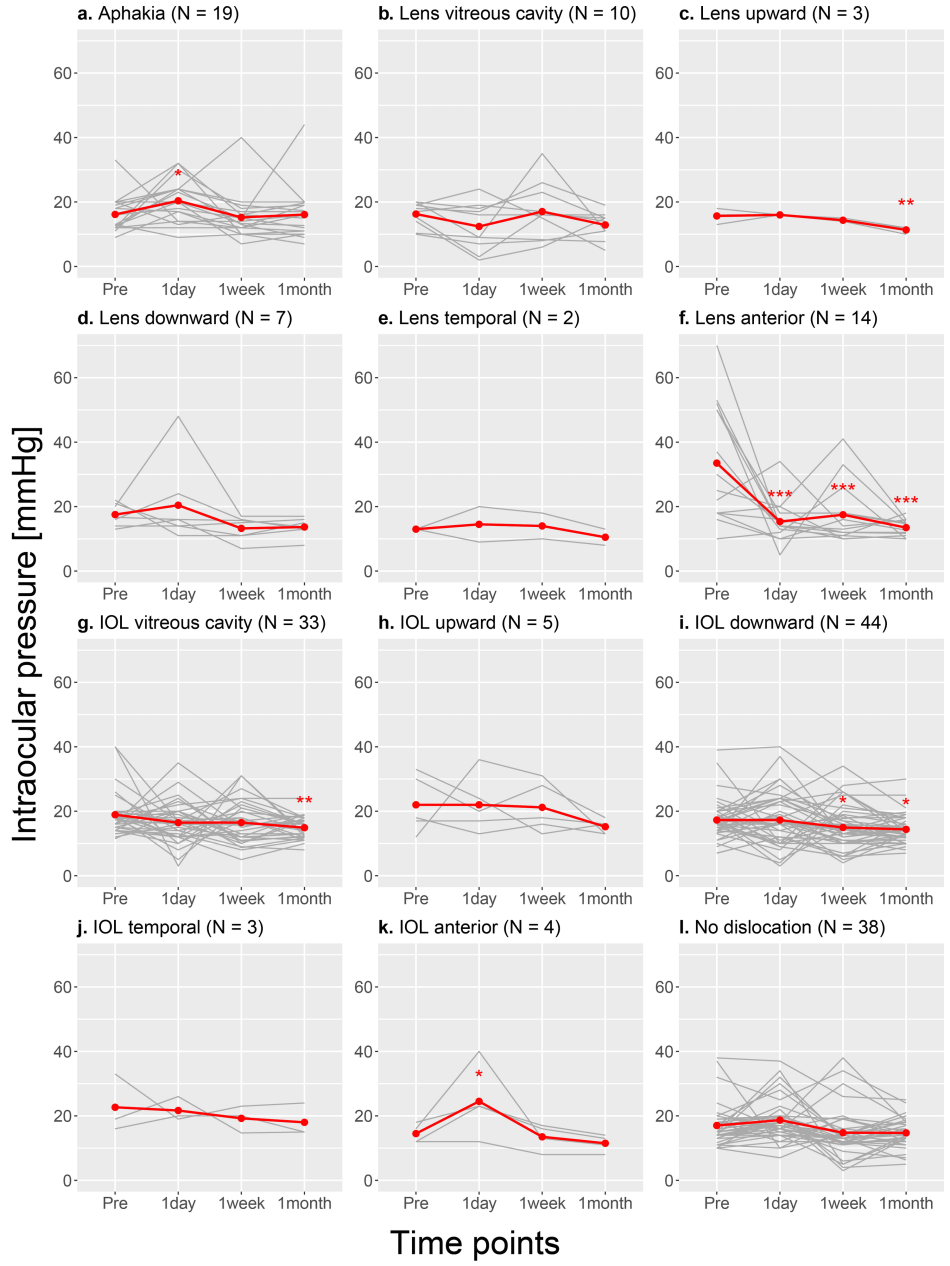
Changes in intraocular pressure preoperatively, one week, and one month postoperatively for each group of the directions of lens and IOL deviation. IOL: intraocular pressure

In the analysis for each group of the directions of lens and IOL deviation, the mean IOP of the cases with anterior lens deviation was significantly higher than that of the other cases before surgery (33.5 mmHg vs. 17.5 mmHg; p<0.001, Mann-Whitney U test), and showed a significant decrease at one day, one week, and one month after surgery (p<0.001, mixed-effect model) (Table 7 and Figure 5).

**Table 7:** Intraocular pressure in relation to the direction of lens/intraocular lens deviation in cases that underwent intrascleral intraocular lens fixation surgery. *The p-value in repeated measures analysis of variance (ANOVA) using mixed-effect model. **P<0.05 was considered significant. ***P<0.001 was considered significant. ****P<0.01 was considered significant. The data were represented as n(%), for the observed number of cases (case or sex), median, IQR for age; and mean±SD for IOP (mmHg). IQR: interquartile range; IOP: intraocular pressure; SD: standard deviation; NumDF: numerator degrees of freedom; DenDF: denominator degrees of freedom; IOL: intraocular lens; Pre: before surgery; After 1 day: 1 day after surgery; After 1 week: 1 week after surgery; After 1 month: 1 month after surgery

Direction of lens or IOL deviation		Case, n (%)	Age, median (IQR)	Sex		IOP (mmHg), mean±SD				p-Value*
				Male, n	Female, n	Pre	After 1 day	After 1 week	After 1 month	
Aphakia		19 (10.3)	76 (70.5-80.5)	14	5	16.1±5.5	20.4±6.6	15.2±6.9	16.1±7.9	0.052
Crystalline lens	Vitreous cavity	10 (5.4)	69.5 (58.5-75.25)	7	3	16.3±3.8	12.4±7.4	17.0±9.0	12.9±4.2	0.162
	Upward	3 (1.6)	57 (50-65)	2	1	15.7±2.5	16.0±0.0	14.3±0.6	11.3±1.2	0.012**
	Nasal	1 (0.5)	62	1	0	18.0	40.0	11.0	13.0	N/A
	Downward	7 (3.8)	67 (54-70.5)	3	4	17.5±3.5	20.4±12.8	13.2±3.6	13.7±2.9	0.129
	Temporal	2 (1.1)	60.5 (51.75-69.25)	2	0	13.0±0.0	14.5±7.8	14.0±5.7	10.5±3.5	0.669
	Anterior	14 (7.6)	57.5 (53.25-67)	8	6	33.5±18.4	15.4±6.7	17.5±9.4	13.5±2.6	<0.001***
IOL	Vitreouscavity	33 (17.9)	60 (49-71)	26	7	18.9±6.7	16.5±6.5	16.5±6.5	14.9±3.4	0.045**
	Upward	5 (2.7)	49 (45-59)	4	1	22.0±9.0	22.0±8.8	21.2±7.9	15.2±2.2	0.431
	Nasal	1 (0.5)	53	1	0	19.3	22.0	18.3	18.3	N/A
	Downward	44 (23.9)	71.5 (57.5-83)	27	17	17.3±6.1	17.3±8.1	15.0±6.7	14.4±4.4	0.017**
	Temporal	3 (1.6)	60 (53.5-67.5)	3	0	22.7±9.1	21.7±3.8	19.2±4.2	18.0±5.2	0.697
	Anterior	4 (2.2)	55 (48.75-63.5)	3	1	14.5±3.0	24.5±11.6	13.5±4.0	11.5±2.6	0.036**
	Posterior	0	N/A	N/A	N/A	N/A	N/A	N/A	N/A	N/A
No dislocation		38 (20.7)	74.5 (63.25-84)	21	17	17.0±6.5	18.7±6.5	14.8±7.4	14.7±4.4	0.002****

The mixed-effect model analysis by the direction of lens and IOL deviation showed that there are also significant decreases in the IOPs at one week and one month after surgery in downward IOL deviation group, and at one month after surgery in upward lens deviation and IOL fall into the vitreous cavity groups (Figure 5).

**Figure 6:**
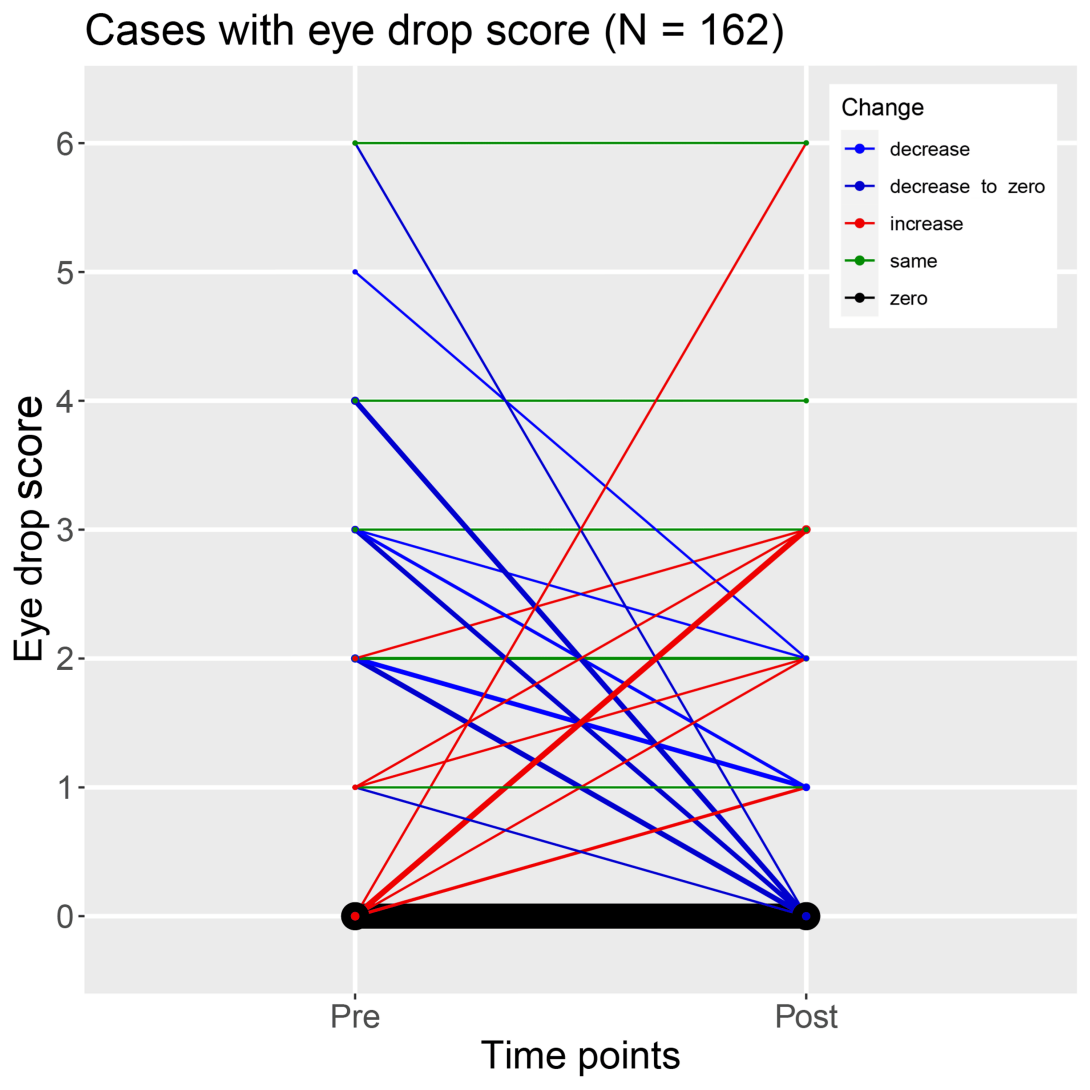
Cases with eye drop score.

In 119 of the 162 cases whose eye drop scores were available, both scores before and after surgery were zero (Table 8 and Figure 6). In 12 cases, the score after surgery decreased to zero from before surgery; in four cases, it decreased but the score after surgery was ≥1; in 15 cases, it increased; in 12 cases, scores before and after surgery were the same (≥1).

**Table 8:** Number of cases devided by eye drop score before and after surgery. The data were represented as "n" for the observed number of cases.

Eye drop score		Pre- and post-change	Cases (n)
Pre	Post		Total (n=162)
0	0	Zero	119
1	0	Decrease to zero	4
2	0	Decrease to zero	5
3	0	Decrease to zero	1
4	0	Decrease to zero	1
6	0	Decrease to zero	1
2	1	Decrease	1
3	1	Decrease	1
3	2	Decrease	1
5	2	Decrease	1
1	1	Same	2
2	2	Same	2
3	3	Same	6
4	4	Same	1
6	6	Same	1
0	1	Increase	5
0	2	Increase	4
0	3	Increase	2
0	6	Increase	1
1	2	Increase	1
1	3	Increase	1
2	3	Increase	1

## Discussion

The indications for IOL intrascleral fixation included long eye axis (20.3%), PEX (18.6%), trouble in surgery (17.8%), history of trauma (16.9%) and so on; however, in 24.6% of cases, surgery was performed for unknown causes. These unknown causes may include causes that were not identified in the interview, but numerous genetic disorders and other causes may have been missed. Some of the cases classified as "unknown" may have reasons other than the diagnoses we have assumed. Some of them may have been overlooked because a detailed interview or family history was not conducted.

In addition, it was observed that 23.7% of cases had multiple diagnoses. Among them, a case involving both PEX and complications during surgery was one of the most common. It was reported that the prevalence of PEX in the Japanese population was 3.4% [11], and it is also known that PEX is one of the causes of intraoperative problems during cataract surgery [12]. Therefore, the observation of many cases with both PEX and trouble in surgery is convincing. The case with both long eye axis and a history of vitrectomy was also one of the most common cases with multiple diagnoses. It was reported that the mean eye axial length for the patients with IOL dislocation after lens replacement surgery was 25.6 mm, so a history of vitrectomy might be relevant to a long eye axis [13]. On the other hand, considering the previous report that 15.2% of junior high school students in Japan have an eye axis length of 26 mm or longer, it seems that the number of persons with a long eye axis is much larger than those with the other diagnoses [14]. However, only 16 of 48 cases with long eye axis had no other diagnosis. This result suggests that a patient with a long eye axis complicated by the other diagnosis may tend to undergo IOL intrascleral fixation more often than with a long eye axis alone, even though a patient with only a long eye axis may necessitate IOL intrascleral fixation.

In the cases of lens deviation, anterior displacement and fall into the vitreous cavity are most often observed in this study. On the other hand, downward displacement and fall into the vitreous cavity are most popular in the case of IOL deviation. Abnormalities in the position of the lens are often caused by trauma. In the trauma cases, the direction of deviation also varied, perhaps because of the various directions of impact. IOL downward deviation and fall into the vitreous cavity were more common in this study, and it is most likely that the weight of the lens placed a strain on the superior ciliary zonule throughout life. In addition, regarding IOL deviation after pars plana vitrectomy, it has been suggested that extensive peripheral vitrectomy probably damages the zonular fibers and results in the dislocation of the IOL [13]. The reason why there are many aphakia cases with PEX or trouble in surgery is that they were referred to our clinic after cataract surgery performed at other hospitals, and intraoperative problems prevented IOL insertion.

In this study, we found that IOP decreased in the short term (one month postoperatively) compared to the preoperative period as a whole. In cases with upward and anterior deviations of the lens, IOL fall into the vitreous cavity and downward deviation of the IOL demonstrated a significant decrease in IOP after surgery, whereas there were no significant differences between the preoperative and postoperative IOP in cases with aphakic eyes and no dislocation.

Regarding the relationship between each factor and IOP changes, significant differences in preoperative and postoperative IOP were observed in the group with a history of vitrectomy and the group with a long eye axis, whereas there were no significant IOP changes in the PEX group or the group with intraoperative problems. This result may reflect the situation in which the IOL fell into the vitreous cavity and dislocated downward more frequently in the group with a history of vitrectomy and the group with a long eye axis, while the PEX group and the intraoperative trouble group had more aphakia cases and cases with no deviation. Significant differences in preoperative and postoperative IOP were also observed in cases with a history of trauma, but the presence of intraocular residuals may play a role in the increase in IOP, as most of the cases showed lens dislocation in a variety of directions. These results support the idea that the presence of intraocular residues influences the increase in IOP.

Crystalline lenses and IOL dislocation in chambers may be linked to hypertension. We hypothesized that the three main factors are circulatory disturbances of the aqueous humor, inflammation, and pigment dispersion. Abnormalities in the position of the lens and IOL may cause retention of aqueous humor, which may inhibit smooth circulation and increase IOP [4]. In particular, we suspect that the extent to which the circulatory disturbances of the aqueous humor are affected by trauma and anterior lens deviation, and the extent to which the trauma causes inflammation, especially if the aqueous humor outflow tract is obstructed, have a strong influence on preoperative high intraocular pressure. In fact, a significantly high preoperative IOP was observed in the cases with anterior lens deviation in this study and surgery resulted in good IOP reduction. Cases with anterior lens deviation are prone to IOP elevation due to pupillary block. In fact, it has been reported that lens subluxation is often misdiagnosed as angle closure glaucoma [15].

Residual lens material in the eye is also known to cause high IOP. The first is phacolytic glaucoma, which occurs when the lens protein itself and the macrophages that phagocytose it obstruct the outflow of aqueous humor [16]. The other is phacoanaphylactic glaucoma, which is secondary to lens-induced uveitis, caused by an immune response to lens proteins [17]. In some cases, including those with intraoperative cataract problems, there may have been residual lens material in the eye, causing the inflammation described above and elevated IOP. Third, we discuss the increase in intraocular pressure caused by pigment dispersion. There is a report that when pigment dispersion was observed in a case in which an intraocular lens was fixed over the lens capsule or in a case in which inadequate intrascleral fixation was performed, the intraocular pressure decreased when intrascleral fixation was performed again. In addition to circulatory disturbance, the presence of pigment dispersion may contribute to intraocular pressure [18,19].

We were able to examine eye drop scores in about 70% of the cases in this study, and in almost all cases, eye drop score was zero both preoperatively and postoperatively. Therefore, the effect of eye drop score on IOP trends in this study is not considered to be very strong. It should be noted that IOL intrascleral fixation does not lower IOP; rather, patients who require IOL intrascleral fixation are likely to have elevated IOP for some background reasons, and intrascleral fixation is not recommended for lowering IOP.

The limitations of this study include the first being related to IOP measurement. The day after surgery, IOP was measured in the ward using GAT; however, in the outpatient setting, both GAT and NCT were used, and measurements were not taken by a single examiner. Nevertheless, GAT and NCT are thought to be correlated [20]. Furthermore, some of the patients considered to be progressing well were referred back soon after the treatment, and it is assumed that their IOPs would not have been high. A possible bias in the results is that if they had been included in the data analysis to the end, the IOP reduction might have been more significant. In addition, some patients originally had glaucoma and were on eye drops and others were on eye drops due to high intraocular pressure caused by lens dislocation; however, it is difficult to account for all of them precisely in this study. Second, long-term changes and the development of complications were not included in this study. In many cases, patients referred for IOL intrascleral fixation were then referred back to the original institution relatively soon after surgery; therefore, long-term follow-up was not available.

The study includes many different factors (direction of the dropped lens, diagnosis previous to surgery, etc.) and there are many limitations as mentioned above. Therefore, absolute conclusions must await prospective studies with large numbers of cases.

## Conclusions

A retrospective study of patients who underwent intrascleral IOL fixation surgery revealed a wide distribution of different causes. On the other hand, about 25% of patients had no known cause. As a whole, surgery reduced IOP in the short term (one month postoperatively). Anterior deviation and fall into the vitreous cavity were more common in the natural crystalline lens eyes, while fall into the vitreous cavity and downward deviation were more common in the IOL eyes. Postoperative IOP was significantly lower in cases of upward, anterior deviation of the lens, fall into the vitreous cavity, and downward deviation of IOL, while there was no significant difference in pre and postoperative IOP in cases of aphakic eyes and no transposition. It was suggested that inflammation due to intraocular remnants and dislocation of the lens and IOL may cause an increase in IOP.
